# ADGRE2: A Promising Immunotherapeutic Target in Acute Myeloid Leukemia and Chronic Myelomonocytic Leukemia

**DOI:** 10.1002/cam4.72497

**Published:** 2026-11-09

**Authors:** Jennifer Neiss, Lisa Hemetsberger, Carolin Schaefer, Marc Rijksen, Antonia Schwab, Armin Zebisch, Andreas Reinisch, Heinz Sill, Annkristin Heine, Albert Wölfler

**Affiliations:** ^1^ Division of Hematology Medical University of Graz Graz Austria; ^2^ Otto‐Loewi‐Research Center for Vascular Biology, Immunology and Inflammation, Division of Pharmacology Medical University of Graz Graz Austria; ^3^ Department of Blood Group Serology and Transfusion Medicine Medical University of Graz Graz Austria

**Keywords:** acute myeloid leukemia, chronic myelomonocytic leukemia, targeted therapy

## Abstract

Acute myeloid leukemia (AML) and chronic myelomonocytic leukemia (CMML) are heterogeneous myeloid malignancies with limited therapeutic options and often poor long‐term prognosis due to persistence of therapy‐resistant leukemic stem cells (LSC). Immunotherapeutic approaches have demonstrated high efficacy in lymphoid malignancies, but their application in AML and CMML remains challenging due to the lack of targetable (neo‐)antigens. Here we investigated the expression of the adhesion G protein‐coupled receptor E2 (ADGRE2) in diagnostic samples from 124 AML and 28 CMML patients using multiparameter flow cytometry and publicly available expression databases. ADGRE2 was significantly overexpressed on leukemic blasts in both AML and CMML compared to normal CD34^+^ hematopoietic stem and progenitor cells (HSPCs). ADGRE2 surface expression was high within the CD34^+^/38^−^ and GPR56^high^ LSC‐enriched compartments, comparably distributed with minor differences among subgroups and high in distinct samples with low expression of established immunotherapeutic targets, such as CD33 and CLL‐1. Our findings further corroborate ADGRE2 as a promising (complementary) immunotherapeutic target in myeloid malignancies.

## 1 Introduction

Acute myeloid leukemia (AML) and chronic myelomonocytic leukemia (CMML) are heterogeneous myeloid malignancies, characterized by the clonal expansion of immature myeloid progenitors and impaired normal hematopoiesis [1, 2]. Despite substantial progress in treatment, the long‐term prognosis for many patients remains poor particularly due to high relapse rates driven by the persistence of therapy‐resistant leukemic stem cells (LSC) [3, 4, 5, 6]. While chimeric antigen receptor (CAR) T‐cell therapies have revolutionized the treatment of lymphoid malignancies, their application in AML and CMML has been limited by a lack of suitable targetable (neo‐)antigens that are both broadly expressed on leukemic cells and absent from healthy hematopoietic stem and progenitor cells (HSPC), thereby minimizing the risk of on‐target off‐tumor toxicity [1, 7, 8, 9, 10]. Consequently, the identification of novel surface antigens with selective expression on leukemic blasts and LSC‐enriched compartments represents a critical step toward advancing immunotherapeutic strategies in these diseases [11].

ADGRE2, also known as CD312 or EGF‐like module‐containing mucin‐like hormone receptor‐like 2 (EMR2), is an adhesion G protein‐coupled receptor that has recently gained attention as a potential surface target for CAR T cell therapies in AML [7]. Initially described in the context of myeloid cell differentiation, maturation, and activation [12], ADGRE2 is increasingly recognized for its potential role in AML pathogenesis, where it may contribute to cellular adhesion, signal transduction, and the maintenance of proteostasis under leukemic stress conditions [13]. Notably, ADGRE2 has also been implicated in glioma and other solid tumors, further supporting its role in malignant transformation and tumor cell persistence [14]. Although its precise function in normal hematopoiesis remains incompletely understood, early evidence suggests that ADGRE2 is aberrantly upregulated in AML patients [15].

Importantly, recent expression profiling efforts have identified ADGRE2 among a shortlist of candidate surface markers with the potential to selectively target leukemic cells while sparing normal HSPC [16, 17]. This is particularly relevant in the context of LSC‐enriched populations, such as the CD34^+^CD38^−^ and the GPR56^+^ compartment, which have been closely linked to disease propagation and treatment resistance [3, 7, 18, 19]. However, a comprehensive characterization of ADGRE2 expression across clinically and genetically defined AML subgroups, especially within leukemic stem cell (LSC)‐enriched compartments, has thus far been lacking.

CMML, while less frequently studied in the context of CAR‐based immunotherapies, shares key biological features with AML, including the presence of clonal blasts and overlapping molecular abnormalities [20]. To date, ADGRE2 expression in CMML has not been systematically investigated and may represent a novel therapeutic target.

Therefore, the aim of this study was to comprehensively characterize ADGRE2 surface expression in diagnostic AML and CMML samples. In particular, we assessed ADGRE2 expression across clinically and genetically defined AML subgroups and within leukemic stem cell (LSC)‐enriched compartments to further evaluate its potential as an immunotherapeutic target.

## 2 Patients and Methods

A total of 124 newly diagnosed AML and 28 CMML patients classified according to the current WHO criteria were included in this study, which was approved by the Institutional Review Board of the Medical University of Graz, Austria (protocol 35–474 ex22/23). Patients were selected based on the availability of diagnostic material suitable for flow cytometric analyses. ADGRE2 surface expression was assessed by multiparameter flow cytometry using the antibody clone 2A1, which selectively targets the extracellular stalk region of ADGRE2 [21, 22], and quantified as both the mean fluorescence intensity (MFI) ratio of the geometric mean and the percentage of ADGRE2‐positive cells [3, 23, 24]. Overall survival (OS) was defined as the time from diagnosis to death from any cause or last follow‐up. Complete remission (CR) was assessed according to standard response criteria and defined as achievement of CR within a maximum of two induction cycles. Sample processing, flow cytometry, and statistical analyses were performed as previously described [3, 23]. Detailed patient characteristics, materials and methodological procedures, as well as the data availability statement are provided in the Supporting Information.

## 3 Results and Discussion

### 3.1 ADGRE2 Expression Is Significantly Increased in AML and CMML Blasts as Compared With Normal CD34 ^+^ HSPCs and Shows Minimal Variation Across (Cyto)genetic Subgroups

Flow‐cytometric expression analysis revealed that ADGRE2 surface levels were markedly increased in AML samples compared to normal CD34^+^ HSPCs. The ADGRE2 MFI ratio was significantly higher in AML blasts (median: 6.239) than in normal HSPCs (3.865; *p* = 0.0303; Figure 1A and Figure S1A). Consistently, the proportion of ADGRE2‐positive cells was significantly increased among AML blasts (median: 89.15%) relative to normal CD34^+^ cells (26.43%; *p* < 0.0001; Figure 1B). Subgroup analysis demonstrated that ADGRE2 expression by MFI was not significantly higher in CD34‐positive AML samples (defined by at least 2% CD34‐expressing leukemic blasts within the bulk leukemia population to exclude major contamination of this population by normal HSPC) compared to CD34‐negative cases (Figure 1D). Accordingly, the overall percentage of ADGRE2‐positive blasts did not significantly differ between these groups (Figure 1F), indicating that ADGRE2 is broadly expressed across immunophenotypic AML subtypes. When stratified by major genetic subgroups, *NPM1*‐mutated samples showed an increased ADGRE2 MFI ratio (median 7.37 vs. 5.301, *p* = 0.002) as well as a higher percentage of ADGRE2‐positive cells (median 95.8% vs. 78.4%, *p* = 0.0014) as compared to wild‐type (wt) samples (Figure 1E,G). No differences were found in FLT3‐mutated, core‐binding factor (CBF) leukemias or AML samples harboring myelodysplasia‐related mutations as compared to wt samples, respectively (Figure 1E,G). Interestingly, when analyzing the BEAT AML cohort, *NPM1*‐mutated samples also displayed higher *ADGRE2* mRNA expression as compared to *NPM1* wt cases (see Figure S2A). Finally, although we noticed subtle differences in *ADGRE2* mRNA expression in the BEAT AML cohort (Figure S2B), we did not observe such differences in samples of our cohort according to ELN risk categories (see Figures S2C and S2D). These findings confirm and extend previous transcriptomic and proteomic studies suggesting that ADGRE2 represents a robustly and broadly expressed surface target for immunotherapeutic strategies in AML [13, 15, 16].

**FIGURE 1:**
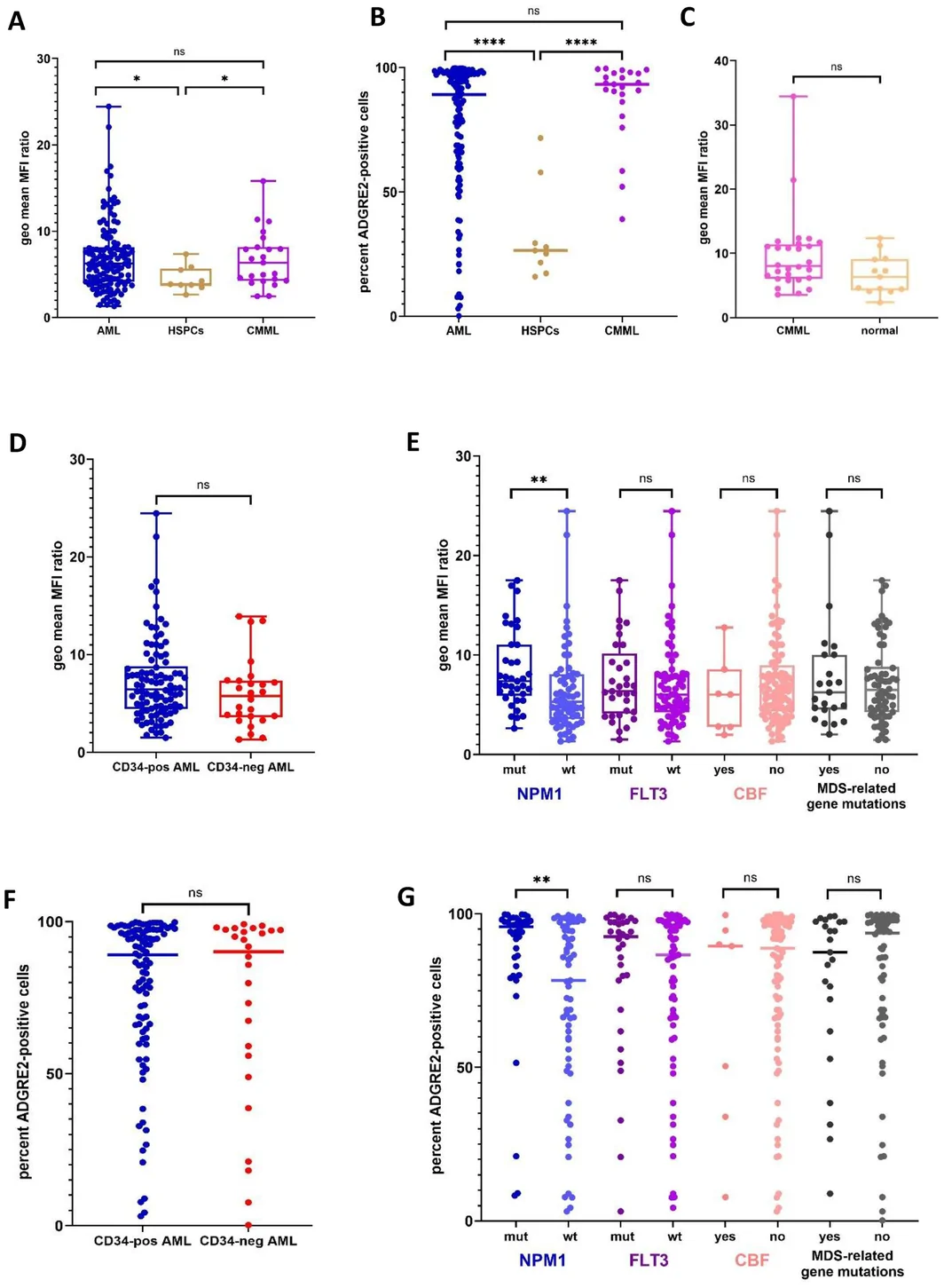
(A) ADGRE2 expression as determined by the MFI ratio displayed as boxplots with the median, the interquartile and the complete range in AML (*n* = 124) and CMML blasts (*n* = 23; 5 samples had to be excluded due to low CD34+ cell counts) as well as normal CD34^+^ HSPCs (*n* = 9). (B) Percentage of ADGRE2‐positive cells among AML (*n* = 124) and CMML blasts (*n* = 23) as well as CD34^+^ HSPCs (*n* = 9) given as dot plot including the median value. (C) ADGRE2 expression in CMML monocytes (*n* = 28) and normal monocytes (*n* = 13). (D) ADGRE2 expression as determined by the MFI ratio in blasts of CD34‐positive (*n* = 100) versus CD34‐negative AML (*n* = 24) samples. (E) ADGRE2 expression as determined by the MFI ratio in blasts in various genetic subgroups of AML. (NPM1 mutated, *n* = 37 vs. NPM1 wildtype (wt), *n* = 63; FLT3 mutated, *n* = 33 vs. FLT3 wt, *n* = 67; core binding factor (CBF) leukemias, *n* = 7 vs. non CBF leukemias, *n* = 93; AML with MDS‐related gene mutations, *n* = 23 vs. AML without MDS‐related gene mutations, *n* = 62). (F) Percentage of ADGRE2‐positive blasts of CD34‐positive vs. CD34‐negative AML samples. (G) Percentage of ADGRE2‐positive blasts in various genetic subgroups of AML. The Mann–Whitney U test was used for all statistical comparisons between groups. (*p < 0.05, **p < 0.01, ***p < 0.001, ****p < 0.0001; ns denotes not significant).

In CMML, CD34^+^ blasts also exhibited significantly elevated ADGRE2 levels compared with normal CD34^+^ HSPCs (*p* = 0.0368 for MFI ratio; *p* < 0.0001 for percentage of positive cells; Figure 1A,B and S1B), closely mirroring the expression pattern seen in AML. ADGRE2 expression on CMML monocytes was comparable to that observed in M1‐monocytes from healthy donors (Figure 1C and Figures S1B and S3). Interestingly, concerning the normal myeloid compartment in peripheral blood, the highest expression of ADGRE2 was found on M2 monocytes, while neutrophils displayed much lower expression levels (Figures S1B and S3).

Owing to the limited number of CMML cases, correlation analyses with specific genetic alterations could not be performed. Nevertheless, to our knowledge, this is the first study reporting elevated ADGRE2 expression in CMML blasts. These results suggest that ADGRE2‐directed therapeutic approaches may extend beyond AML to other myeloid neoplasms with overlapping blast cell phenotypes.

### 3.2 LSC‐Enriched Compartments Display High Levels of ADGRE2 Surface Expression but Expression Patterns Do Not Affect Overall Survival in Intensively Treated AML Patients

To represent a clinically useful target for immunotherapeutic strategies in AML, a surface marker must also be robustly expressed within LSC‐enriched populations, such as the CD34^+^CD38^−^ and the GPR56^+^ compartment, which are critically involved in disease propagation and treatment resistance [3, 7, 18, 19]. In CD34‐positive AML samples (*n* = 100), ADGRE2 expression levels and the fraction of positive cells were comparably high in the CD34^+^/38^−^ LSC‐enriched compartment and the more differentiated CD34^+^/38^+^ fraction (Figure 2A,B). In contrast, CD34^−^ blasts within CD34‐positive AML samples exhibited significantly lower ADGRE2 expression levels and reduced percentages of ADGRE2‐positive cells compared with the CD34^+^ subpopulations (*p* < 0.001 for MFI ratio; *p* < 0.0001 for percentage; Figure 2A,B).

**FIGURE 2:**
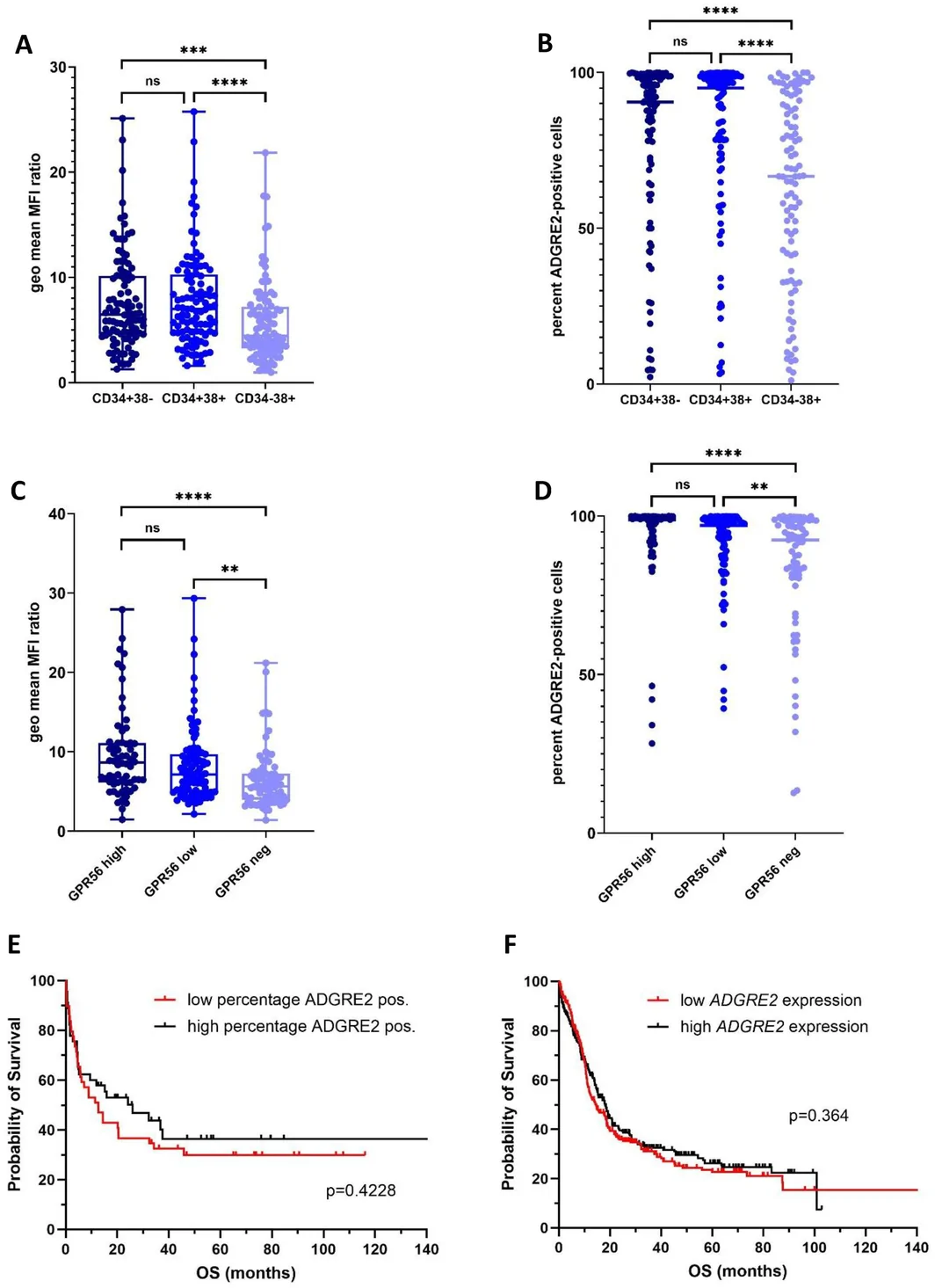
(A) ADGRE2 expression as determined by the MFI ratio displayed as boxplots with the median, the interquartile and the complete range in subpopulations of CD34‐positive AML samples. (B) Percentage of ADGRE2‐positive cells among subpopulations of CD34‐positive AML samples given as dot plot including the median value. (C) ADGRE2 expression as determined by the MFI ratio in GPR56 subpopulations of AML samples (*n* = 88). (D) Percentage of ADGRE2‐positive cells among GPR56 subpopulations of AML samples (*n* = 88). The Mann–Whitney U test was used for all statistical comparisons between groups. (E) Overall survival in AML patients receiving intensive chemotherapy (*n* = 64) according to their ADGRE2 surface expression (each group *n* = 32). (F) Overall survival in patients in the BEAT AML cohort receiving therapy (*n* = 888) according to their *ADGRE2* expression (each group *n* = 444). Survival curves were generated using the Kaplan–Meier method, and differences between groups were evaluated using the log‐rank test. (**p < 0.01, ***p < 0.001, ****p < 0.0001; ns denotes not significant).

As a complementary strategy to define LSC‐enriched AML subpopulations, we employed GPR56 expression, as AML cells with high GPR56 levels are enriched for LSC irrespective of their CD34‐status [3, 7, 18, 19]. Both, ADGRE2 expression intensity and the proportion of positive cells were highest in the LSC‐enriched GPR56^high^ compartment and lowest in GPR56^−^ AML blasts (Figure 2C,D). Accordingly, when we analyzed publicly available data [25], *ADGRE2* mRNA expression was higher in AML LSC than in AML blasts. Low expression levels for *ADGRE2* expression were found in normal HSPC (see Figure S4). These results indicate that ADGRE2 (surface) expression is not only elevated in the bulk leukemic population but is also strongly found within immunophenotypically defined LSC‐containing compartments. This is highly relevant, as therapeutic failure in AML is frequently attributed to the persistence of LSCs, which are relatively quiescent and may show resistance to both cytotoxic and targeted treatments [3, 4, 5, 6].

As a minority of samples in our cohort exhibited relatively low ADGRE2 expression, combinatorial targeting of multiple surface antigens may enhance the efficacy of CAR T‐ cell therapy in AML while potentially reducing toxicity [24]. Haubner et al. demonstrated that cooperative targeting of ADGRE2 together with additional antigens, like CLL‐1, improved AML cell elimination while limiting antigen escape [7]. To assess whether ADGRE2 may provide complementary target coverage in AML, we analyzed 25 AML samples for which surface expression data for CD33, CLL‐1, and CD117 were also available [23]. Comparison of the proportions of AML blasts expressing these established target antigens with those expressing ADGRE2 revealed a trend toward a positive correlation between the percentages of CD117‐positive and ADGRE2‐positive AML cells (Figure S5A). Accordingly, only 2 of 25 samples showed a low proportion (< 60%) of CD117‐positive blasts together with a high proportion (> 80%) of ADGRE2‐positive blasts. In contrast, 10 of 25 samples (40%) exhibited a low proportion of CLL‐1‐positive blasts combined with a high proportion of ADGRE2‐positive blasts (Figure S5B). This pattern was even more pronounced for CD33, with 12 of 25 samples (48%) showing a low proportion of CD33‐positive blasts but a high proportion of ADGRE2‐positive blasts (Figure S5C). These findings suggest that ADGRE2 may provide complementary target coverage in a subset of AML cases with relatively limited expression of established targets such as CD33 or CLL‐1. Together with the strong and stable expression of ADGRE2 across LSC‐enriched compartments, its complementary expression pattern further supports the rationale for combinatorial targeting strategies. However, given that ADGRE2 expression has also been detected at low levels on normal HSPCs and at higher levels on mature myeloid cells, both in our study (Figure 1C and Figures S1 and S3) and in previous reports [7, 12, 21, 25, 26], careful assessment of potential on‐target/off‐tumor toxicity will be essential before clinical translation.

Finally, we investigated whether ADGRE2 expression levels influence clinical outcome in AML. Overall survival (OS) was analyzed in all treated patients (*n* = 94), stratified by high versus low ADGRE2 expression using a cut‐off of 89% ADGRE2‐positive blasts (median value of the cohort). No statistically significant differences in OS were observed between the two groups (Figure 2E). Complete remission (CR) rates were analyzed separately in patients receiving intensive chemotherapy (*n* = 64), as CR represents a clinically meaningful endpoint in this treatment setting. CR was defined as achievement of complete remission within a maximum of two induction cycles. Likewise, CR rates did not differ between patients with high and low ADGRE2 expression (75.8% vs. 74.2%). Accordingly, OS was not affected by ADGRE2 expression levels in the BEAT AML cohort (Figure 2F).

Our findings are in line with previous studies demonstrating increased *ADGRE2* expression in AML compared with normal hematopoietic cells [15]. However, unlike Yang et al. [15], who reported an association between increased *ADGRE2* expression and inferior survival in AML, we did not observe a significant association between ADGRE2 protein surface expression and either overall survival or complete remission rates in our cohort. These differences may be explained by variations in patient cohorts, treatment regimens, and analytical methods. In particular, Yang et al. analyzed *ADGRE2* gene expression, whereas our study assessed ADGRE2 protein surface expression by flow cytometry, which is directly relevant for evaluating ADGRE2 as a potential immunotherapeutic target.

The strengths of our study include the comprehensive flow cytometric analysis of ADGRE2 surface expression in a relatively large cohort of AML patients, the inclusion of CMML samples, and the detailed characterization of leukemic stem cell‐enriched compartments. The main limitations of this study are its retrospective single‐center design, the relatively small CMML cohort, and the lack of experimental validation of ADGRE2 as a therapeutic target. Therefore, our findings warrant further validation in independent cohorts and future preclinical studies investigating ADGRE2‐targeted immunotherapeutic approaches.

In summary, our study identifies ADGRE2 as a consistently and robustly expressed surface molecule in AML and CMML cells and LSC‐enriched compartments. These findings provide a strong rationale for further preclinical and translational evaluation of ADGRE2 as an immunotherapeutic target, either as a single agent or in combination with additional antigens for the treatment of myeloid malignancies.

## Author Contributions

**Jennifer Neiss:** conceptualization, investigation, methodology, data curation, formal analysis, writing – original draft. **Lisa Hemetsberger:** investigation, writing – review and editing, methodology, formal analysis. **Carolin Schaefer:** investigation, writing – review and editing, methodology, formal analysis. **Marc Rijksen:** writing – review and editing, formal analysis, validation, methodology, software. **Antonia Schwab:** investigation, writing – review and editing, methodology. **Armin Zebisch:** writing – review and editing, resources, formal analysis. **Andreas Reinisch:** resources, writing – review and editing, formal analysis. **Heinz Sill:** resources, writing – review and editing, formal analysis. **Annkristin Heine:** writing – review and editing, resources, formal analysis. **Albert Wölfler:** data curation, supervision, project administration, writing – original draft, conceptualization, methodology, validation, formal analysis, funding acquisition, visualization.

## Funding

This work was supported by Leukämiehilfe Steiermark and Austrian Science Fund, P36672, PAT1753824, I6101.

## Ethics Statement

Written informed consent was obtained from all patients prior to sample collection. The study was approved by the Institutional Review Board of the Medical University of Graz, Austria (protocol 35–474 ex22/23).

## Conflicts of Interest

The authors declare no conflicts of interest.

## Supporting information


**Table S1:** Patient Characteristics.
**Table S2:** Antibodies used for multiparameter flow cytometry.
**Figure S1:** Flow Cytometry Gating strategy.
**Figure S2:** ADGRE2 expression in AML subgroups.
**Figure S3:** ADGRE2 surface expression in normal myeloid cells in peripheral blood.
**Figure S4:** ADGRE2 expression in normal and leukemic stem and progenitor cells.
**Figure S5:** Correlation of ADGRE2 surface expression with other targets used in immuno‐therapeutic approaches in AML, such as CD33, CD117 and CLL‐1.

## Data Availability

All data generated and analyzed during this study are available from the corresponding author upon reasonable request and after completion of a Data Transfer Agreement.

## References

[1] S. Haubner , M. Subklewe , and M. Sadelain , “Honing CAR T Cells to Tackle Acute Myeloid Leukemia,” Blood 145, no. 11 (2025): 1113–1125, 10.1182/blood.2024024063. 39630061

[2] A. M. Hunter , M. M. Patnaik , R. Itzykson , et al., “Perspectives on Drug Development in Chronic Myelomonocytic Leukemia: Changing the Paradigm,” Blood 144, no. 19 (2024): 1987–1992, 10.1182/blood.2024025648. 39509115

[3] S. Daga , A. Rosenberger , F. Quehenberger , et al., “High GPR56 Surface Expression Correlates With a Leukemic Stem Cell Gene Signature in CD34‐Positive AML,” Cancer Medicine 8, no. 4 (2019): 1771–1778, 10.1002/cam4.2053. PMC6488118 30848055

[4] D. Thomas and R. Majeti , “Biology and Relevance of Human Acute Myeloid Leukemia Stem Cells,” Blood 129, no. 12 (2017): 1577–1585, 10.1182/blood-2016-10-696054. PMC5364335 28159741

[5] D. Hanekamp , B. Denys , G. J. L. Kaspers , et al., “Leukaemic Stem Cell Load at Diagnosis Predicts the Development of Relapse in Young Acute Myeloid Leukaemia Patients,” British Journal of Haematology 183, no. 3 (2018): 512–516, 10.1111/bjh.14991. 29076143

[6] P. Stelmach and A. Trumpp , “Leukemic Stem Cells and Therapy Resistance in Acute Myeloid Leukemia,” Haematologica 108, no. 2 (2023): 353–366, 10.3324/haematol.2022.280800. PMC9890038 36722405

[7] S. Haubner , J. Mansilla‐Soto , S. Nataraj , et al., “Cooperative CAR Targeting to Selectively Eliminate AML and Minimize Escape,” Cancer Cell 41, no. 11 (2023): 1871–1891.e6, 10.1016/j.ccell.2023.09.010. PMC11006543 37802054

[8] R. Shao , Z. Li , H. Xin , et al., “Biomarkers as Targets for CAR‐T/NK Cell Therapy in AML,” Biomarker Research 11, no. 1 (2023): 65, 10.1186/s40364-023-00501-9. PMC10276424 37330575

[9] T. Haslauer , R. Greil , N. Zaborsky , and R. Geisberger , “CAR T‐Cell Therapy in Hematological Malignancies,” International Journal of Molecular Sciences 22, no. 16 (2021): 8996, 10.3390/ijms22168996. PMC8396650 34445701

[10] P. Vishwasrao , G. Li , J. C. Boucher , D. L. Smith , and S. K. Hui , “Emerging CAR T Cell Strategies for the Treatment of AML,” Cancers 14, no. 5 (2022): 1241, 10.3390/cancers14051241. PMC8909045 35267549

[11] S. Saito and Y. Nakazawa , “CAR‐T Cell Therapy in AML: Recent Progress and Future Perspectives,” International Journal of Hematology 120, no. 4 (2024): 455–466, 10.1007/s12185-024-03809-w. 38963636

[12] G. W. Chang , J. Q. Davies , M. Stacey , et al., “CD312, the Human Adhesion‐GPCR EMR2, Is Differentially Expressed During Differentiation, Maturation, and Activation of Myeloid Cells,” Biochemical and Biophysical Research Communications 353, no. 1 (2007): 133–138, 10.1016/j.bbrc.2006.11.148. 17174274

[13] D. Huang , Z. Yu , H. Lu , et al., “Adhesion GPCR ADGRE2 Maintains Proteostasis to Promote Progression in Acute Myeloid Leukemia,” Cancer Research 84, no. 13 (2024): 2090–2108, 10.1158/0008-5472.can-23-2314. 39082681

[14] B. Im , A. Costachi , D. Ao , M. Ev , and O. Zlatian , “Research Progress of EMR2 Receptor Function in Glioma and Its Potential Application as Therapeutic Target,” Current Health Sciences Journal 50, no. 4 (2024): 478–487, 10.12865/CHSJ.50.04.02. PMC11936079 40144939

[15] J. Yang , S. Wu , and H. Alachkar , “Characterization of Upregulated Adhesion GPCRs in Acute Myeloid Leukemia,” Translational Research 212 (2019): 26–35, 10.1016/j.trsl.2019.05.004. PMC7473775 31153896

[16] F. Perna , S. H. Berman , R. K. Soni , et al., “Integrating Proteomics and Transcriptomics for Systematic Combinatorial Chimeric Antigen Receptor Therapy of AML,” Cancer Cell 32, no. 4 (2017): 506–519.e5, 10.1016/j.ccell.2017.09.004. PMC7025434 29017060

[17] R. Shahswar , A. Kloos , R. Gabdoulline , et al., “Frequency and Clinical Characteristics Associated With Putative CAR Targets ADGRE2, CCR1, CD70, and LILRB2 in Acute Myeloid Leukemia,” Blood 132, no. Supplement 1 (2018): 5259, 10.1182/blood-2018-99-113037.

[18] C. Pabst , A. Bergeron , V. P. Lavallée , et al., “GPR56 Identifies Primary Human Acute Myeloid Leukemia Cells With High Repopulating Potential In Vivo,” Blood 127, no. 16 (2016): 2018–2027, 10.1182/blood-2015-11-683649. 26834243

[19] A. Waclawiczek , A. M. Leppä , S. Renders , et al., “Combinatorial BCL2 Family Expression in Acute Myeloid Leukemia Stem Cells Predicts Clinical Response to Azacitidine/Venetoclax,” Cancer Discovery 13, no. 6 (2023): 1408–1427, 10.1158/2159-8290.CD-22-0939. PMC10236156 36892565

[20] H. Kao , M. Kuo , C. Ou , et al., “Clonal Dynamics of Chronic Myelomonocytic Leukemia Progression: Paired‐Sample Comparison,” Journal of Pathology 265, no. 4 (2025): 437–447, 10.1002/path.6396. 39905935

[21] M. J. Kwakkenbos , G. W. Chang , H. H. Lin , et al., “The Human EGF‐TM7 Family Member EMR2 Is a Heterodimeric Receptor Expressed on Myeloid Cells,” Journal of Leukocyte Biology 71, no. 5 (2002): 854–862. PubMed PMID: 11994511, 10.1189/jlb.71.5.854. 11994511

[22] G. W. Chang , M. Stacey , M. J. Kwakkenbos , J. Hamann , S. Gordon , and H. H. Lin , “Proteolytic Cleavage of the EMR2 Receptor Requires Both the Extracellular Stalk and the GPS Motif,” FEBS Letters 547, no. 1–3 (2003): 145–150, 10.1016/S0014-5793(03)00695-1. 12860403

[23] S. Daga , A. Rosenberger , K. Kashofer , et al., “Sensitive and Broadly Applicable Residual Disease Detection in Acute Myeloid Leukemia Using Flow Cytometry‐Based Leukemic Cell Enrichment Followed by Mutational Profiling,” American Journal of Hematology 95, no. 10 (2020): 1148–1157, 10.1002/ajh.25918. PMC7540028 32602117

[24] S. Haubner , F. Perna , T. Köhnke , et al., “Coexpression Profile of Leukemic Stem Cell Markers for Combinatorial Targeted Therapy in AML,” Leukemia 33, no. 1 (2019): 64–74, 10.1038/s41375-018-0180-3. PMC6326956 29946192

[25] A. J. Gentles , “Association of a Leukemic Stem Cell Gene Expression Signature With Clinical Outcomes in Acute Myeloid Leukemia,” Journal of the American Medical Association 304, no. 24 (2010): 2706, 10.1001/jama.2010.1862. PMC4089862 21177505

[26] S. E. Boyden , A. Desai , G. Cruse , et al., “Vibratory Urticaria Associated With a Missense Variant in *ADGRE2* ,” New England Journal of Medicine 374, no. 7 (2016): 656–663, 10.1056/nejmoa1500611. PMC4782791 26841242

