# Supplementary material for: ADGRE2: A Promising Immunotherapeutic Target in Acute Myeloid Leukemia and Chronic Myelomonocytic Leukemia
Source: Cancer Med. 2026 Nov 9;15(11):e72497. doi: 10.1002/cam4.72497 (PMC13746257; doi:10.1002/cam4.72497)
Supplement: Supplementary file 1 [file CAM4-15-e72497-s001.docx]

**Supporting Information**

**ADGRE2: A Promising Immunotherapeutic Target in Acute Myeloid Leukemia and Chronic Myelomonocytic Leukemia**

Jennifer Neiss^1^, Lisa Hemetsberger^1^, Carolin Schaefer^1^, Marc Rijksen^1^, Antonia Schwab^1^, Armin Zebisch^1,2^, Andreas Reinisch^1,3^, Heinz Sill^1^, Annkristin Heine^1^ and Albert Wölfler^1^

^1^ Division of Hematology, Medical University of Graz, Austria

^2^ Otto-Loewi-Research Center for Vascular Biology, Immunology and Inflammation, Division of Pharmacology, Medical University of Graz, Austria

^3^ Department of Blood Group Serology and Transfusion Medicine, Medical University of Graz, Austria

Corresponding author:

Albert Wölfler, MD

Division of Hematology,

Medical University of Graz,

Auenbruggerplatz 38, 8036 Graz, Austria

Tel: +43 316 385 84084

Fax: +43 316 385 14087

Email: albert.woelfler@medunigraz.at

**Content:**

Description of Patients and Methods	2

Table S1: Patient Characteristics	3

Table S2: Antibodies used for multiparameter flow cytometry	4

Figure S1: Flow Cytometry Gating strategy	5

Figure S2: ADGRE2 expression in AML subgroups	7

Figure S3: ADGRE2 surface expression in normal myeloid cells in peripheral blood	8

Figure S4: ADGRE2 expression in normal and leukemic stem and progenitor cells	9

Figure S5: Correlation of ADGRE2 surface expression with other targets used in immuno-

therapeutic approaches in AML, such as CD33, CD117 and CLL-1	10

**Patients and Methods**

**Patients**

In total, 124 newly diagnosed patients with AML and 28 patients with CMML were included in this study. All diagnoses were established in accordance with the current World Health Organization (WHO) classification of myeloid neoplasms. Patient characteristics are summarized in Supporting Information, Table S1. Bone marrow (BM) or peripheral blood (PB) samples were collected at the Medical University of Graz, provided by the Biobank of the Medical University of Graz (Cohort 6002 14, Myeloid Neoplasias Collection) and processed as previously described (1). To assess normal CD34⁺ hematopoietic stem and progenitor cell (HSPC) compartments, BM samples from nine lymphoma patients without any evidence of BM involvement were used. Thirteen normal PB samples obtained from healthy donors served as controls for normal monocytes. Clinical and (cyto)genetic data were retrieved from the hospital electronic documentation system MEDOCS (Medical Documentation and Communication System; SAP, Sinsheim, Germany). Written informed consent was obtained from all patients prior to sample collection. The study was approved by the Institutional Review Board of the Medical University of Graz, Austria (protocol 35-474 ex22/23).

**Flow cytometry analysis**

Multiparameter flow cytometry was performed using a Navios® or Cytoflex cytometer (Beckman Coulter, Brea, CA) with strictly harmonized baseline settings. At time of analysis, cryopreserved mononuclear cells were thawed, washed with phosphate-buffered saline, and stained with the appropriate antibodies (see supporting information, Table S2). At least 100 000 events were recorded, and data were analyzed using Kaluza C 1.2 software (Beckman Coulter, Brea, CA). Appropriate isotype controls were used to determine the level of background staining.

Leukemic blast cells were identified based on low expression of CD45 and low side scatter as well as expression of CD34 and/or CD117 (for gating see supporting information, Figure S1A and S1B). In CD34-positive AMLs, CD38 was used to further define the LSC-containing compartment as CD34^+^/CD38^low/-^. For identification of GPR56^+^ LSC, CD117^+^ leukemic blasts were analyzed for their GPR56-expression and categorized into GPR56^-^, GPR56^low^, or GPR56^high^ cells. For each population, the percentage of ADGRE2-positive cells and the mean fluorescence intensity (MFI) of ADGRE2 expression were recorded. MFI values were calculated as the ratio of the geometric MFI of the specific antibody to that of the corresponding isotype control (see also Figure S1A and S1B).

**Statistical analysis**

Differences in patient characteristics were assessed using two-sided Fisher’s exact test or the Mann–Whitney U test, as appropriate. Comparisons between two groups were performed using the Mann–Whitney U test, while comparisons among more than two groups were conducted using the Kruskal–Wallis test. Survival curves were generated using the Kaplan–Meier method, and differences between groups were evaluated using the log-rank test. All statistical analyses were performed using GraphPad Prism software version 10.6 (GraphPad Software, La Jolla, CA) and SPSS Statistics version 24.0 (IBM, Armonk, NY). All hypothesis tests were two-sided, with a significance level of α = 0.05.

**Data Availability**

All data generated and analyzed during this study are available from the corresponding author upon reasonable request and after completion of a Data Transfer Agreement. Publicly available datasets used in this study were obtained from GEO at GSE24006 and analyzed using gene expression commons (https://gexc.riken.jp/). BEAT AML data were accessed via cBioPortal (<https://www.cbioportal.org/>).

**Table S1: Patient characteristics**

| **AML** (124 patients) | |
| --- | --- |
| **Gender** (female/male) | 53 / 71 |
| **Age** (median, range) | 65 years; 23 – 92 years |
| **Treatment** |  |
| Intensive treatment | 64 |
| Non-intensive treatment | 30 |
| Supportive care only or unknown | 30 |
| **Genetic Aberrations** (mutated/wildtype/unknown) |  |
| *NPM1* | 37 / 67 / 20 |
| *FLT3* | 33 / 67 / 24 |
| Core binding factor - leukemia | 7 / 93 / 24 |
| Myelodysplasia-related mutations | 23 / 62 /39 |
| **ELN risk stratification** (favourable/intermediate/adverse)  unknown | 19 / 44 / 34  27 |
| **CMML** (28 patients) | |
| **Gender** (female/male) | 11 / 17 |
| **Age** (median, range) | 72 years; 42 – 89 years |
| **Genetic Aberrations** (mutated/wildtype/unknown) |  |
| *RAS* signaling pathway mutations | 16 / 3 / 9 |
| *TET2* | 5 / 14 / 9 |
| *SRSF2* | 9 / 10 / 9 |

**Table S2: Antibodies used for multiparameter flow cytometry**

| **CD marker** | **Clone** | **Fluorochrome** | **Company** |
| --- | --- | --- | --- |
| ADGRE2 (CD312) | REA302 | FITC | Miltenyi Biotec |
| CD14 | HCD14 | PerCPcy5.5 | Biolegend |
| CD16 | 3G8 | APC-A750 | Beckman Coulter |
| CD34 | 581 | ECD | Beckman Coulter |
| CD38 | HB-7 | APC | Becton Dickinson |
| CD45 | 2D1 | APC-H7 / V500C | Becton Dickinson |
| CD117 | 95C3 | PC7 / PE | Beckman Coulter |
| GPR56 | CG4 | PE | Biolegend |
| 7-AAD |  |  | Beckman-Coulter |

**Figure S1A: Gating strategy for analysis of CD312-expression in CD34^+^ HSPCs and AML blasts**

**normal bone marrow:**

**MNC	MNC	CD34^+^ cells**

**AML:**

**MFI ratio: geometric MFI CD312/geometric MFI isotype: 412.52/117.88 = 3.50**

**AML 1:**

**MNC	MNC	CD34^+^ cells**

**MFI ratio: 16.60**

**AML 2:**

**MNC	MNC	CD117^dim/-^ blasts**

**CD117**

**MFI ratio: 4.19**

**Figure S1B: Gating strategy for analysis of CD312-expression in CMML blasts and PB myeloid cells**

**CMML:**

**MNC	MNC	CD34^+^ cells**

**MFI ratio: 4.46**

**normal peripheral blood:**

 **cells	granulocytes	CD16^+^ granulocytes**

**CD16**

**MFI-ratio: 2.38**

 **monocytes	M1 monocytes	M2 monocytes**

**MFI ratio: 10.19**

**MFI ratio: 32.92**

**CD14**

**CD16**

**Figure S2: *ADGRE2* expression in AML subgroups**

**B**

**A**

**C**

**D**

**Figure S2:** A) *ADGRE2* mRNA log2 expression levels shown in a violin plot in *NPM1*-mutated (mut, n=220) vs. wildtype (wt, n=661) AML samples from the BEAT AML cohort. B) *ADGRE2* mRNA log2 expression levels shown in a violin plot of samples from the BEAT AML cohort according to their ELN 2017 risk classification (favourable, n=215; intermediate, n=185; adverse, n=259). C) ADGRE2 surface protein expression as determined by the MFI ratio displayed as boxplots with the interquartile and the complete range in samples of our AML cohort according to their ELN 2022 risk classification (favourable, n=19; intermediate, n=44; adverse, n=34). D) Percentage of ADGRE2-positive cells among ELN 2022 risk subgroups of AML samples given as dot plot including the median value. (** p<0.01, *** p<0.001, **** p<0.0001; ns denotes not significant)

**Figure S3: ADGRE2 surface expression in normal myeloid cells in peripheral blood**

**B**

**A**

**Figure S3:** A) ADGRE2 expression as determined by the MFI ratio displayed as boxplots with the median, the interquartile and the complete range in monocyte subtypes and granulocytes of normal healthy donors (n=13). B) Percentage of ADGRE2-positive cells in monocyte subtypes and granulocytes of normal healthy donors (n=13) given as dot plot including the median value. The Mann-Whitney-U test was used for all statistical comparisons between groups. (**** p<0.0001; ns denotes not significant)

**Figure S4: *ADGRE2* expression in normal and leukemic stem and progenitor cells.**

**Figure S4:** *ADGRE2* mRNA expression levels in normal bone marrow (BM) and cord blood (CB) HSPCs as well as AML cells obtained from publicly available microarray data from GEO at GSE24006 analyzed using gene expression commons (<https://gexc.riken.jp/>). HSC denotes hematopoietic stem cells, MPP multipotent progenitors, CMP common myeloid progenitors, MEP megakaryocyte-erythrocyte progenitors, GMP granulocyte-monocyte progenitors; LSC leukemic stem cells, LPC leukemic progenitor cells.

**Figure S5:**	**Correlation of ADGRE2 surface expression with other targets used in immuno-therapeutic approaches in AML, such as CD33, CD117 and CLL-1**

**A**

Pearson r=0.3299, p=0.1039

high (>80%) for ADGRE2, but low (<60%) for CD117:

2 out of 25 (8%)

**B**

Pearson r=0.0639, p=0.7612

high (>80%) for ADGRE2, but low (<60%) for CLL-1:

10 out of 25 (40%)

**C**

Pearson r=0.1223, p=0.5602

high (>80%) for ADGRE2, but low (<60%) for CD33:

12 out of 25 (48%)

**Figure S5:** Correlation of the percentage of ADGRE2-positive cells with CD117- (A), CLL-1- (B) and CD33-positivity (C) in 25 AML samples. The red box represents the area with a high percentage (>80%) of ADGRE2-positive cells but a low percentage (<60%) of cells positive for the respective marker.
